# *Uranotaenia unguiculata* Edwards, 1913 are attracted to sound, feed on amphibians, and are infected with multiple viruses

**DOI:** 10.1186/s13071-018-3030-2

**Published:** 2018-08-06

**Authors:** Jeremy V. Camp, Tamás Bakonyi, Zoltán Soltész, Thomas Zechmeister, Norbert Nowotny

**Affiliations:** 1 Viral Zoonoses, Emerging and Vector-Borne Infections Group, Institute of Virology, University of Veterinary Medicine, Vienna, Austria; 2 Department of Microbiology and Infectious Diseases, University of Veterinary Medicine, Budapest, Hungary; 3 Lendület Ecosystem Services Research Group, MTA Centre for Ecological Research, Vácrátót, Hungary; 4 Hungarian Natural History Museum, Budapest, Hungary; 5 Biological Station Lake Neusiedl, Illmitz, Austria; 6 Department of Basic Medical Sciences, College of Medicine, Mohammed Bin Rashid University of Medicine and Health Sciences, Dubai, United Arab Emirates

**Keywords:** *Pelophylax*, Sound attraction, West Nile virus, *Alphamesonivirus*, Mosquito ecology, Ectothermic hosts

## Abstract

**Background:**

*Uranotaenia unguiculata* Edwards, 1913 is a species of mosquito (Diptera: Culicidae) native to central Europe. Recently a novel lineage of the West Nile virus (WNV-lineage 4c) was identified in pools of adult female *Ur. unguiculata*. To increase the body of knowledge about this species, various trapping methods were evaluated to determine the most efficient method for capturing adult female *Ur. unguiculata*.

**Results:**

Sound traps collected equivalent numbers of female *Ur. unguiculata* as low-hanging light-baited downdraft traps. Hosts were identified as *Pelophylax lessonae* and *P. ridibunda* (Anura: Ranidae) species group frogs from the blood found in engorged females. In addition to confirming infection by WNV-lin. 4c, a potentially integrated flavivirus sequence was detected in male mosquitoes. A novel *Alphamesonivirus 1* (*Nidovirales*: *Mesoniviridae*) was found to be widespread in the *Ur. unguiculata* population and is herein described.

**Conclusions:**

Efficient collection methods for *Ur. unguiculata* for arbovirus surveillance reflect mosquito questing behavior. *Uranotaenia unguiculata* targets frog species which call from the water, and it is likely that the novel WNV-lin. 4c is maintained in a frog-mosquito transmission cycle. The improved trapping methods listed here will assist future studies of the vector status of *Ur. unguiculata* for WNV and other arboviruses.

**Electronic supplementary material:**

The online version of this article (10.1186/s13071-018-3030-2) contains supplementary material, which is available to authorized users.

## Background

*Uranotaenia unguiculata* Edwards, 1913 (Diptera: Culicidae) is a species of mosquito native to the Western Palaearctic with species abundance highest in the Mediterranean biogeographical region [1, 2]. European populations are infrequently collected during arbovirus surveillance programs using conventional mosquito trapping methods (e.g. CO_2_-baited light traps), often comprising less than 0.5% of the total collections [3–6]. It is often reported that *Ur. unguiculata* feeds exclusively on amphibians [1], similar to other members of the genus *Uranotaenia* [7, 8], although what little evidence exists for this behavior in *Ur. unguiculata* is conflicting [9–11]. Comparatively little is known about the mosquito, particularly its importance as a vector of zoonotic viruses.

In 2013, our group reported the existence of a novel lineage of West Nile virus (*Flaviviridae*, “WNV”) in *Ur. unguiculata* from Austria [5], and a similar virus was reported from *Ur. unguiculata* populations in Romania [6] and Hungary [4]. The virus was closely related to WNV-lineage 4 (WNV-lin. 4a) found in Russia in *Ur. unguiculata* [12] and in Spain in *Culex pipiens* (Linnaeus, 1758) (Diptera: Culicidae) (WNV-lin. 4b) [13]. Investigations at a study site in Volgograd, Russia, identified virus nucleic acid (WNV-lin. 4a) in both the frog population [*Pelophylax ridibundus* (Pallas, 1771) (= *Rana ridibunda* Pallas, 1771)] (Anura: Ranidae) as well as the *Ur. unguiculata* population [14]. However, no study has conclusively determined that *Ur. unguiculata* feed on frogs. Furthermore, nucleic acid from a potentially unique flavivirus has been identified from a population of *Ur. unguiculata* in Turkey [15]. Therefore, the potential of *Ur. unguiculata* to vector arboviruses to humans and other animals remains unknown.

Herein we describe improved trapping methods to target the collection of adult *Ur. unguiculata* at a site in the Pannonian biogeographical region of central Europe. We sought to improve upon standard mosquito trapping methods, using both modified traditional and non-conventional mosquito traps, to collect adult females. A longitudinal study was performed using the improved method over a single collection season, yielding many individuals, including males and blood-engorged female specimens. As a result*,* we provide further support to the hypothesis that WNV-lin. 4c is transmitted to frogs by *Ur. unguiculata*, and describe a novel alphamesonivirus isolated from a pool of male *Ur. unguiculata*.

## Methods

### Study site

The majority of mosquito collections were performed at Lake Neusiedl, the largest endorheic lake in central Europe. Mosquito trapping was focused at the Biological Station Lake Neusiedl, Illmitz (“BSI,” 47°46.12'N, 16°45.69'E) in 2016, as well as sites near the towns of Winden am See, Purbach am Neusiedler See, and Breitenbrunn (centered approx. around 47°55.90'N, 16°44.78'E) on the western shore of the lake in 2017. Approximately 240 km^2^ (76%) of the shallow steppe lake lies in the eastern Austrian federal state of Burgenland, and the remainder in Hungary, in the western Pannonian biogeographical region. The lake reaches only 1.8 m in depth (mean and SD of daily water quality measurements taken at BSI from June-September, 2016–2017: 2062 ± 240 μS/cm^2^, pH 8.9 ± 0.1), is surrounded by extensive vegetation [*Phragmites* sp. (Poaceae)], and supports diverse avian and amphibian assemblages. A single trapping session in Hungary was performed at a site near Kajászó (47°18.86'N, 18°40.98'E) in August 2017.

### Mosquito collection

Adult mosquitoes were collected with modified CDC Light Traps (John W. Hock Co. Gainesville, FL, USA) using either fluorescent or ultraviolet lights. Traps were placed 1 m from the water edge and the trap intake was 0.5 m from the water surface, and run from 1 h before sunset until 1 h after sunrise. The sampling session in Hungary was performed with a strong mercury lamp over a one hour period approximately 1 h after sunset (21:00–22:00 h). Resting adults were collected using a backpack aspirator (John W. Hock Co, Gainesville, FL, USA) from fixed sites at BSI sampled routinely in the morning and evening: pathways through the reeds cleared by semiaquatic rodents, a man-made wooden boardwalk extending above water into the reeds from shore, and four black plastic refugia (30 × 30 cm boxes, 15 cm tall, lacking a western-facing side, similar to [16]) which were placed at various sites near the water.

Sound traps were modified from gravid traps (John. W. Hock Co. Gainesville, FL, USA): a black plastic basin was filled with 2 cm lake water and an updraft fan was positioned above the water. A small 3 cm speaker was placed at the mouth of the fan intake and broadcast the recorded call of individual *Dryophytes gratiosus* (LeConte, 1856) (Anura: Hylidae), a species of tree frog native to the southeastern United States whose call is attractive to Neotropical *Uranotaenia* species [17]. The sound of the calling male frog consisted of approximately one call per s for 14 s, and was broadcast repeatedly for 2 h beginning 1 h after sunset, setting the volume at maximum each night (Additional file 1: Figure S1). Mosquitoes were anesthetized by 5 min incubation at -20 °C and sorted to species on an ice-cold plate according to morphologic characters described in [1]. Samples were pooled (*n* < 50 per pool) by species, sex, and date and stored at -80 °C until analysis.

### Vertebrate host identification

DNA was extracted from individually separated abdomens of blood-fed specimens (the remaining body parts were pooled by date for virus analysis) using a commercial kit (DNEasy, Qiagen GmbH, Hilden, Germany). Hosts were identified by PCR following published methods which use vertebrate-specific primers designed to amplify portions of the mitochondrial gene *16S* rRNA (“L2513”, 5'-GCC TGT TTA CCA AAA ACA TCA C-3'; “H2714”; 5'-CTC CAT AGG GTC TTC TCG TCT T-3') [18] or *cytochrome b* (5'-CCC CTC AGA ATG ATA TTT GTC CTC A-3'; 5'-GCH GAY ACH WVH HYH GCH TTY TCH TC-3') [7]. The amplicons were subjected to Sanger sequencing (Microsynth AG, Balgach, Switzerland), and sequences were compared to voucher specimens collected from the study site (kindly provided by Silke Schweiger, curator of the herpetology collection of the Austrian Museum of Natural History, Vienna, Austria).

### Virus detection using RT-qPCR

A single copper-coated steel bead was added to each pool of mosquitoes, and the pools were homogenized in virus growth media [VGM, composed of Dulbecco’s minimum essential medium (DMEM), supplemented with 10% fetal calf serum (FCS), penicillin/streptomycin, and 0.25 μg/ml amphotericin B, all cell culture reagents from Gibco, ThermoFisher Scientific, Paisley, UK] using a TissueLyzer bead mill with a pre-cooled rack set to 30 Hz for 1 min (Qiagen GmbH, Hilden, Germany). Homogenate was cleared by centrifugation at 8000× *g* for 4 min at 4 °C, and supernatant was stored at -80 °C. Total RNA was extracted from the pellet using a commercial kit (Zymo Research Corp., Irvine, CA, USA). A one-step RT-qPCR assay was performed with universal flavivirus primers (PF1S and PF2) targeting a portion of the flavivirus NS5 [19] using a commercial kit (Luna®, New Enlgand Biolabs, Inc., Ipswitch, MA, USA, “NEB”). A second RT-qPCR was used to confirm putative identifications, using pan-flavivirus primers (100F and 200R) and a commercial kit (NEB) [20]. Using a probe-based RT-qPCR kit (NEB) alphamesonivirus nucleic acid was detected in mosquito pools with primers designed to match a conserved portion of the ORF1b putative replicase domain (MesoF, 5'-ACC GGC CTT GCA CAT CTA AA-3'; MesoR, 5'-CGC GGG TAG GTT TCA GTG TA-3'; MesoP, 5'-6-carboxyfluorescein [FAM]-AGA CAA CTT AGC GGT GTG GA-black hole quencher 1 [BHQ1]-3').

### Virus rescue and identification of unknown virus

Virus was rescued from putative positive homogenates on C6/36 insect cells (ATCC #CRL-1660). Briefly, C6/36 cells were incubated with 100 μl of homogenate on a 6-well plate. After 1 h, DMEM with 2% FCS, antibiotics, and antimycotics were added to each well. On day 6 post-infection, cell culture supernatant was blind-passaged into new C3/63 cells and to Vero cells (ATCC #CCL-81). When cytopathic effect (CPE) was observed, supernatant was filtered through 0.2 μm filter and purified through a 36% sucrose cushion at 28000× *rpm* in a cooled ultracentrifuge. The pellet was treated with RNase and DNase (Promega) for 1 h at 37 °C and RNA was extracted from the pellet as described above. First and second strand cDNA were synthesized with 40U AMV reverse transcriptase (Promega, Mannheim, Germany) followed by treatment with 1U RNase H and 20U Klenow fragment DNA polymerase (Promega) using non-specific primers with a known sequence at the 5' end (5'-GAC CAT CTA GCG ACC TCC ACN NNN NNN N-3') as described by others for the sequence-independent amplification of virus particle-associated nucleic acids (PANA) [21]. The cDNA was used as a template for PCR using primers for the known sequence (5'-GAC CAT CTA GCG ACC TCC AC-3'), and the resulting amplicons were TA-cloned into a pGEM vector (Promega). Cloned inserts ≥ 500 bp were detected by colony PCR using M13 primers and *Taq* polymerase (GoTaq G2® DNA polymerase, Promega) and amplicons were sequenced. These sequences were compared to sequences in the GenBank database using the basic local alignment search tool (BLAST), and primers were designed from the closest-matching sequences to produce a near full-length viral sequence by primer-walking (Additional file 2: Table S1). The sequence was deposited in the GenBank database under the accession number MH215275.

### Sequence characterization of Alphamesonivirus 1 isolate

The near full-length sequence (missing portions of the 3' and 5' sequence) of the alphamesonivirus isolate was aligned to reference sequences from the family *Mesoniviridae* using the MUSCLE algorithm, and sequence analyses were performed in MEGA 6.06 [22, 23]. Percent sequence identity for aligned nucleotide and amino acid sequences were calculated with the “Sequence Manipulation Suite” [24]. A maximum likelihood (ML) tree was constructed from the amino acid sequence alignment for the open reading frame encoding the conserved putative spike protein (ORF 2a). The initial tree was obtained by the neighbor-joining method. The final tree was generated using the Jones-Taylor-Thornton matrix-based model with a very strong branch swap filter, and ML estimates are based on bootstrap resampling of 1000 replicates.

### Determination of mosquito infection and virus transmission

In June 2018, *Ur. unguiculata* mosquitoes were collected using sound traps. Individuals were held for 2–5 days at ambient temperature and natural light in humidified chambers and provided 25% solution of local honey in water on Whatman® FTA® cards. The honey cards were changed daily and stored at -80 °C thereafter. The legs and wings were removed from mosquitoes and stored in 250 μl VGM in pools of 1–10 individuals. The rest of the mosquito (head, abdomen and thorax) was stored in 500 μl VGM in pools of 2–50 individuals. Pooled mosquito parts were homogenized using a bead mill, and RNA was extracted as described above. If virus nucleic acid was detected in a pool of mosquito bodies by RT-PCR methods described above, then the corresponding pools of legs and wings were similarly tested for the presence of virus nucleic acid. The presence of virus nucleic acid in the legs and wings indicates a disseminated infection. To test for virus transmission, RNA was extracted from the FTA honey cards by first soaking the card in 500 μl Tris-EDTA buffer for 1 h with shaking, then extracting RNA from 200 μl of the Tris-EDTA solution as described above.

### Statistical analysis

A two-tailed binomial test was used to compare the collection efficiency of trap methods with the null hypothesis that traps collect equal numbers of mosquitoes. A sign test was used to compare between methods over paired trap-nights. The minimum field infection rate (MFIR) was calculated with ML estimator statistics using “Pooled infection rate 7.0” Microsoft Excel plug-in available from the United States Centers for Disease Control and Prevention, according to methods described therein [25]. Figures were prepared in GraphPad Prism5.

## Results

### Summary of collection methods for *Uranotaenia unguiculata*

**Fig. 1:**
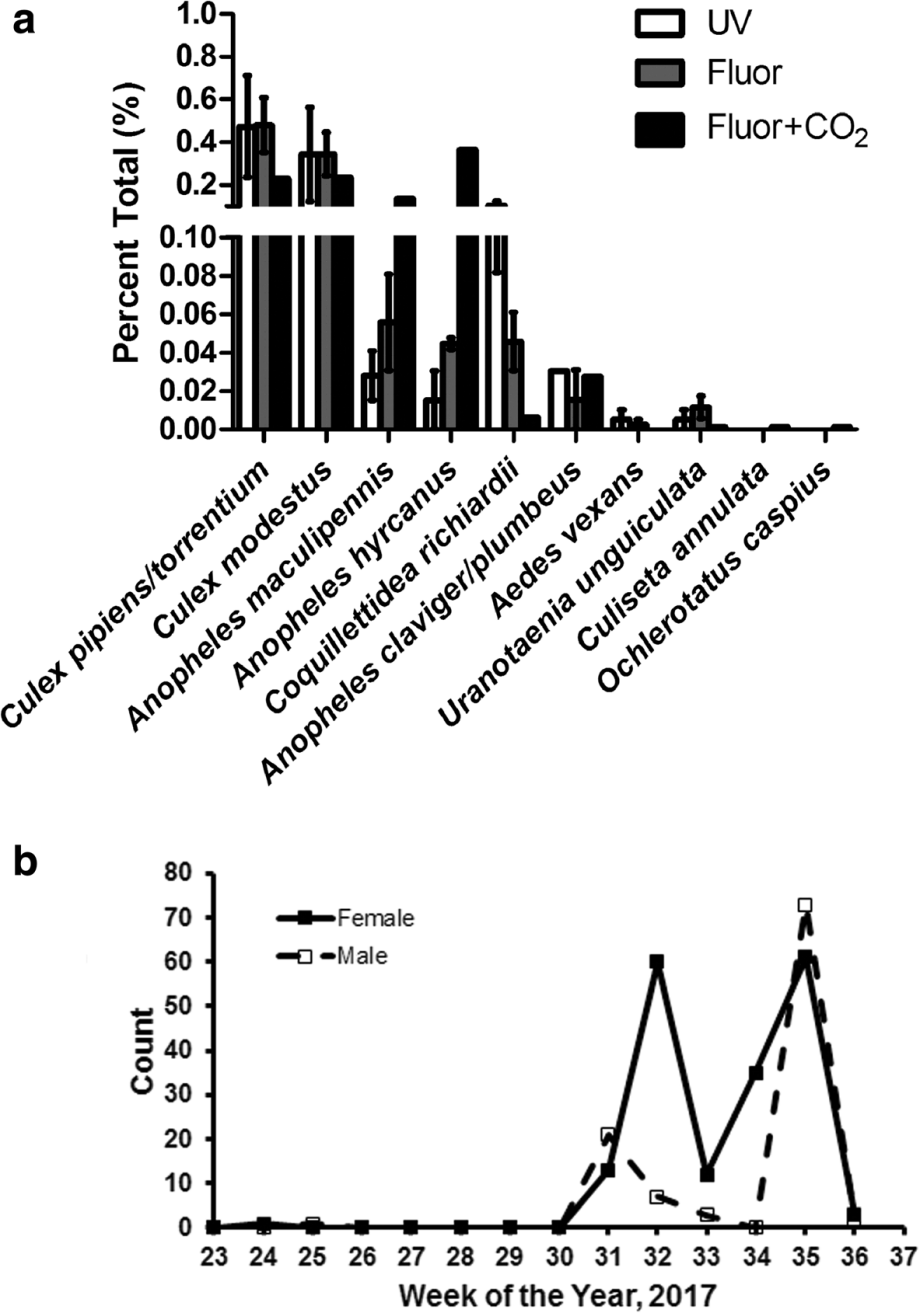
Mosquito collections at Lake Neusiedl in eastern Austria. **a** The average percent (± SEM) of mosquito species collected in CDC light traps with a UV light (*n* = 6 trap-nights), a fluorescent light (“Fluor”, *n* = 4 trap-nights), or a fluorescent light in combination with a source of CO_2_ (“Fluor+CO_2_”, *n* = 2 trap-nights); traps were paired from August-September 2016. **b** Total *Uranotaenia unguiculata* (females, closed symbols and solid line; males, open symbols and dashed line)

From 9 August 2016 to 15 September 2016, miniature CDC light traps were used at BSI to collect *Ur. unguiculata* in order to establish a baseline collection rate. Traps were baited with a fluorescent light (*n* = 4 trap-nights), UV light (*n* = 6 trap-nights), or fluorescent light with dry ice as a source of CO_2_ (*n* = 2 trap-nights). In total, 3347 mosquitoes (3025 females) were collected over 12 trap-nights. Both males (9.6% of the total collection) and females of *Anopheles* sp. (Diptera: Culicidae), *Coquillettidia richiardii* Ficalbi, 1899 (Diptera: Culicidae), *Culex* sp. (Diptera: Culicidae), and *Ur. unguiculata* were captured in all light traps. *Culex pipiens*, *Cx. torrentium* Martini, 1925 (Diptera: Culicidae) (46.7%, *Cx. pipiens* were not differentiated from *Cx. torrentium*) and *Cx. modestus* Ficalbi, 1889 (Diptera: Culicidae) (12.5%) were the dominant culicine mosquito species collected at the site, whereas *An. hyrcanus* (Pallas, 1771) (30.5%) and *An. maculipennis* Meigen, 1818 (*s.l.*) (Diptera: Culicidae) (5.9%) were the dominant anopheline species (Fig. 1a). More male *Ur. unguiculata* (*n* = 274) were collected than females (*n* = 108, average 8.5/trap-night, SD = 12.1). Significantly more *Ur. unguiculata* were captured at fluorescent-baited traps than UV (39%) traps (binomial test, *P* = 0.011; sign test for trap-night, *P* = 1.00), although trap success was low until mid-September. Due to low collection size, the difference in collection efficiency of *Ur. unguiculata* between traps baited with and without CO_2_ could not be inferred. However, the addition of CO_2_ appeared to have an effect on other species (e.g. fewer *Cq. richiardii* and more *Anopheles* sp. were collected in traps with CO_2_ than without, Fig. 1a).

No *Ur. unguiculata* were collected from artificial resting boxes nor from natural resting sites in reeds. On 12 September 2017, 13 male and 2 female *Ur. unguiculata*, which appeared to be newly emerged imagoes, were captured by aspiration resting under a boardwalk that extended over shallow water into the reeds. Larvae were present in the water beneath this collection site.

These preliminary findings represented a substantial increase in the number and proportion of *Ur. unguiculata* in comparison to previous similar trapping methods: here we report 3.6% of total female mosquitoes collected were *Ur. unguiculata*, whereas others report *Ur. unguiculata* made up < 1% of total collections in this region [5, 26]. Therefore bi-weekly trapping was performed with a single low-hanging fluorescent light trap placed as before at BSI beginning 31 May 2017. A single female *Ur. unguiculata* was captured on 14 June 2017 and a single male *Ur. unguiculata* was captured on 22 June 2017. Following that, no *Ur. unguiculata* were captured until August, and the peak collection period began on 2 August 2017 and continued until 7 September 2017 (Fig. 1b).

To test the hypothesis that *Ur. unguiculata* are attracted to sound, sound traps were used at several locations around the lake. Each night a sound trap was used, a ‘mock’ sound trap (updraft trap with no sound) was placed 3–10 m away, and a fluorescent light trap was placed at the same site > 10 m from the sound trap and out of sight from the sound trap (*n* = 5 nights). No *Ur. unguiculata* were captured in ‘mock’ sound traps, whereas 86 female *Ur. unguiculata* were captured in the sound traps (average 12.2 per trap-night; range 0–36) (Table 1). The trap success of the sound traps was not different from light traps (compared on *n* = 5 nights, average 12.4 per trap-night in light traps; range 1–27) (binomial test, *P* = 0.06; sign test for differences in trap night, *P* = 1.00) (Table 1).

**Table 1:** Collections of *Uranotaenia unguiculata* using sound traps (Sound +/-) and light traps (LT)

Trap night	Sound +	Sound -	LT
8 August 2017	36	–	7
9 August 17	10	0	–
16 August 2017	4	0	1
21 August 2017	26	0	1
28 August 2017	0	0	26
29 August 2017	6	–	27
7 September 2017	4	0	–
Total	86	0	62
Mean	12.3		12.4
SD	13.4		13.1

### Hosts of *Uranotaenia unguiculata*

Eight blood-engorged female *Ur. unguiculata* were collected from 8 August 2017 through 7 September 2017: five individuals from sound traps, and three individuals from light traps (including two from Hungary captured using a mercury lamp). Hosts were identified from the blood meal using *16S* rRNA PCR [18], and amplicons were compared to a DNA library made from voucher specimens. All were identical to *Pelophylax lessonae*/*ridibundus* species group voucher specimens collected from the site. These identifications were confirmed using *cytb* PCR protocol [7]. Although voucher specimens exist for *P. lessonae*, *P. ridibundus* and *P. esculentus* (which make up the species group), these species could not be differentiated by the sequenced amplicons.

### Flavivirus identification

In 2016, 108 female and 274 male *Ur. unguiculata* were captured and divided into 11 and 10 pools, respectively; and in 2017, 185 female *Ur. unguiculata* and 107 males were captured and divided into 14 and 7 pools, respectively, for flavivirus screening. Two pools of females and one pool of males from 2017 tested positive for the presence of flavivirus nucleic acids (*NS5* gene) by both RT-qPCR methods (Table 2). Sequencing of these amplicons showed that the pools of females (2017, MFIR = 12; 95% CI: 2.2–40.8) were positive for WNV-lin. 4c with 99% sequence identity to published sequences from 2013 (KJ891223 “WNV Uu-LN-AT-2013”) [4, 5], differing in only 3 synonymous nucleotide substitutions across the 205 bp product of the *NS5* gene; and 99% sequence identity to an 800 bp portion of the *E* gene, differing at 3 synonymous nucleotide substitutions. A bias-corrected ML estimator calculation for the MFIR was 12.05 (95% CI: 2.20–40.76) for female *Ur. unguiculata* over both years.

**Table 2:** Summary of arboviruses identified in *Uranotaenia unguiculata* mosquitoes collected from Austria, 2016–2017

Sex	Virus	2016		2017	
		Total	Pools	Total	Pools
Female		108	11	185	14
	*WNV-lin. 4c*		0		2
	*Alphamesonivirus*		5		7
Male		274	10	107	7
	*WNV-lin. 4c*		0		0
	*Alphamesonivirus*		5		2

The flavivirus RNA-positive pool of males displayed closest sequence identity (100%) to a published sequence that was detected in *Ur. unguiculata* from Turkey (GenBank: KU958167) [15]. The presence of the DNA form of the sequence was confirmed by PCR amplification from the RNA extract (i.e. without reverse transcription).

### Alphamesonivirus isolation and characterization

**Fig. 2:**
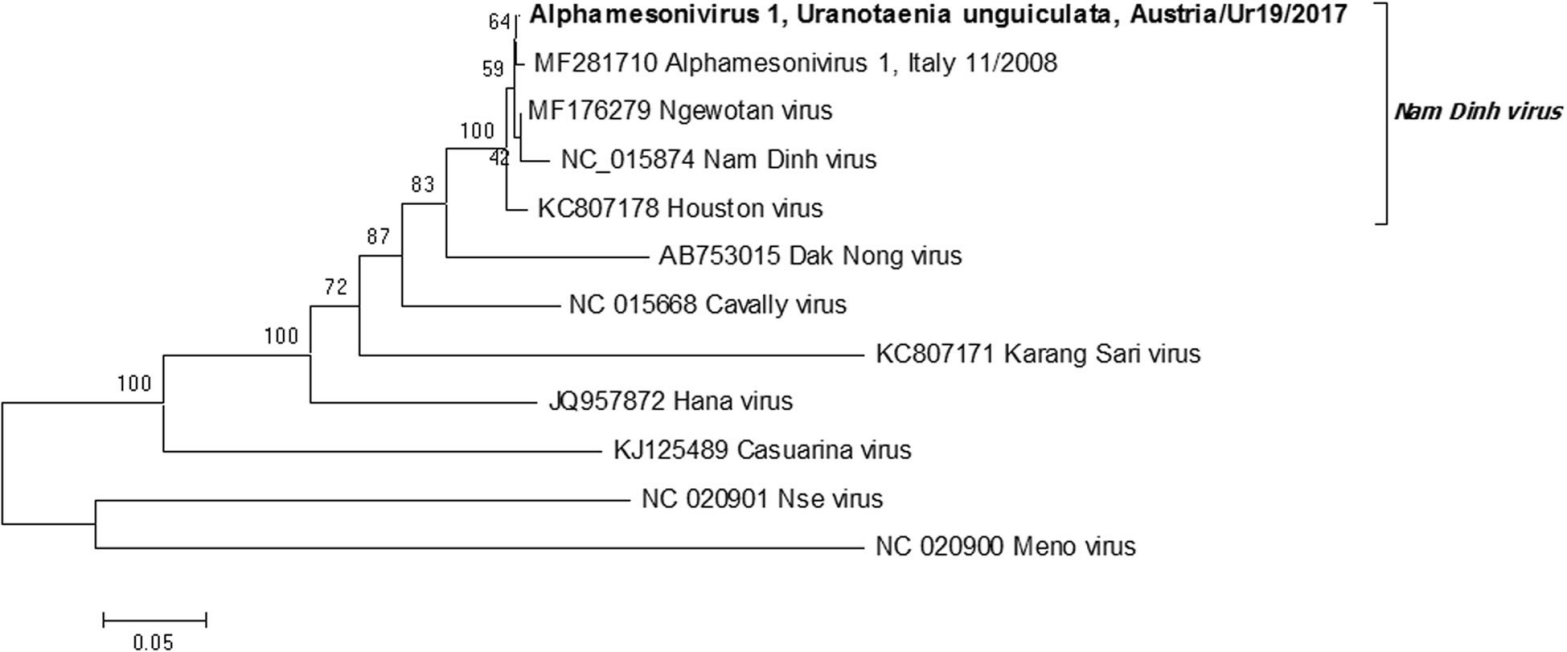
Phylogenetic tree of the amino acid sequence of the putative spike protein (complete ORF2a) from selected species of *Mesoniviridae*, including a newly described isolate from *Uranotaenia unguiculata* in Austria (MH215275). Node support is based on 1000 bootstrap replicates

Two pools of male *Ur. unguiculata* had putative positive amplification by one flavivirus RT-qPCR (where *Ct* > 30 but melting curve analysis did not match positive controls) [19]. Therefore the pool homogenates was inoculated onto C6/36 cells. One pool caused CPE at 6 days post-infection, and was then filtered and passed to flasks of C6/36 cells. This passage (p2) was used for sequence characterization by PANA [21], as it did not produce amplicons by flavivirus-specific RT-qPCR. This passage did not produce CPE on Vero cells grown at 37 °C. Following PANA PCR cloning, the virus was determined to be an alphamesonivirus, based on the sequence of 6 unique gene products approximately 500–2000 bp long distributed throughout the genome. Primers were designed based on sequence similarity to previously characterized alphamesoniviruses (Additional file 2: Table S1), and PCR amplicons were sequenced to create a nearly complete genome sequence (missing approximately 300 bases from the 5' and 50 bases from the 3' end) (GenBank: MH215275). A phylogenetic tree of the alignment of the conserved putative spike protein (ORF 2a) showed that the isolated virus was closely related to Nam Dinh virus (GenBank: DQ458789), placing it within the species group *Alphamesonivirus 1*, and more distantly related to the following representative species of the Mesoniviridae: *Alphamesonivirus 2,* Karang Sri virus (GenBank: KC807171); *Alphamesonivirus 3*, Dak Nong virus (AB753015); *Alphamesonivirus 4*, Casuarina virus (GenBank: KJ125489); *Alphamesonivirus 5*, Hana virus (GenBank: JQ957872); *Mesonvirus 1*, Nse virus (GenBank: JQ957874); and *Mesonivirus 2*, Meno virus (GenBank: JQ957873) (Fig. 2). The virus sequence had all features associated with the genus *Alphamesonivirus*, including the reported ribosomal frame-shift site separating ORF1a and ORF1b, which encode the putative viral replicase [27, 28]. The virus isolate had the highest amino acid and nucleotide sequence identity (99.33 and 99.41%, respectively) to an alphamesonivirus identified in *Cx. pipiens* in Italy, 2008 (GenBank: MF281710, Table 3).

**Table 3:** Sequence identity matrix of species of *Mesoniviridae*, including a new isolate in *Uranotaenia unguiculata* from Austria

	NaDV	NgeV	Houston NaDV	CaV	Austria NaDV	Italy NaDV	KSV	DNV	CasV	HanaV	NseV	MenoV
Nam Dinh virus (NaDV) (NC_015874)		98.8	96.8	90.5	**97.7**	97.6	78.4	86.9	74.8	84.8	68.5	64.2
Ngewotan NaDV (NgeV) (MF176279)	98.3		97.6	90.8	**98.5**	98.3	78.6	87.3	74.8	85.2	68.4	63.9
Houston NaDV (KC807178)	96.8	98.2		90.6	**98.6**	98.5	78.4	87.0	74.5	84.9	68.1	63.6
Cavally virus (CaV, NC_015668)	87.7	88.2	87.8		**90.4**	90.5	74.9	84.2	74.6	84.0	67.1	63.8
**Austria NaDV (MH215275)**	**97.9**	**99.3**	**98.2**	**87.9**		**99.4**	**78.3**	**86.9**	**74.4**	**84.9**	**68.2**	**63.6**
Italy NaDV (MF281710)	97.9	99.1	98.0	88.2	**99.3**		78.2	87.0	74.4	85.0	68.1	63.6
Karang Sari virus (KSV, KC807171)	74.3	74.7	74.8	72.4	**74.4**	78.2		75.7	68.2	75.5	63.9	60.8
Dak Nong virus (DNV, AB753015)	86.7	87.6	87.1	82.8	**87.3**	87.3	72.2		73.9	81.34	67.6	63.6
Casuarina virus (CasV, NC_023986)	70.2	70.2	70.2	69.6	**70.15**	70.3	62.5	69.8		73.6	66.0	63.2
Hana virus (HanaV, NC_020899)	82.1	83.0	82.8	80. 9	**83.0**	83.0	71.3	79.9	69.6		67.5	64.3
Nse virus (NseV, NC_020901)	63.0	63.0	62.9	61.6	**63.2**	63.4	57.3	62.7	59.4	63.4		62.0
Meno virus (MenoV, NC_020900)	58.0	57.9	57.9	56.8	**58.0**	57.9	54.2	57.2	55.7	57.3	56.6	

Percent nucleotide sequence identity (above the diagonal) and percent amino acid identity (below the diagonal) are shown (Austrian isolate in bold). GenBank accession numbers are listed in the row headings, and column heading abbreviations are defined in the row heading

An RT-qPCR assay was designed to screen the other pools of male and female mosquitoes for the presence of alphamesonivirus. Ten pools from 2016 (five pools of females) and nine pools from 2017 (seven pools of females) were positive for alphamesonivirus (Table 2). A bias-corrected ML estimate for MFIR was 17.3 (95% CI: 7.89–35.28) for males and 54.19 (95% CI: 33.87–86.92) for female *Ur. unguiculata* over both years.

### Mosquito infection and virus transmission

A total of 362 female *Ur. unguiculata* mosquitoes were collected over four nights around Lake Neusiedl in June 2018. The legs and wings were dissected and the remaining bodies were pooled in 44 pools for the detection of virus (Table 4). The presence of WNV-lin. 4c was detected in nine of these pools, corresponding to 225 mosquitoes from which legs and wings had been pooled into 39 pools. WNV-lin. 4c was detected in six of the 39 pools. The potential for virus transmission was determined by detecting virus nucleic acid on honey-soaked FTA cards which had been placed into chambers with mosquitoes for feeding purposes prior to their dissection. WNV-lin. 4c nucleic acid was detected on one of nine honey cards, suggesting that *Ur. unguiculata* are capable of transmitting the virus. Alphamesonivirus was detected in three pools of mosquito bodies, corresponding to 11 mosquitoes, from which the legs and wings had been placed into tubes individually. From these 11 mosquitoes, alphamesonivirus was detected in the legs and wings of two individuals, indicating a disseminated infection. The presence of alphamesonivirus nucleic acid was detected on three of the nine honey cards, suggesting the presence of the virus in saliva.

**Table 4:** Summary of infection and potential transmission of West Nile virus lineage 4c and *Alphamesonivirus 1* by *Uranotaenia unguiculata* mosquitoes

Virus	Sample^a^	Total	Pools^b^	Positive pools^c^
West Nile virus	Body	362	44	9
	Legs and wings	225	39	6
	Honey card	-	9	1
*Alphamesonivirus 1*	Body	362	38	3
	Legs and wings	11	11	2
	Honey card	-	9	3

^a^Mosquitoes were provided a 25% honey solution on Whatman® FTA® cards, then dissected, removing legs and wings from the body

^b^Legs and wings were stored separately from body, and samples were pooled into tubes of 2–50 (bodies) or 1–10 (legs and wings). Each honey card sampled between 20-50 mosquitoes

^c^Pools were tested for the presence of virus by RT-PCR

## Discussion

Herein we report the first molecular identification of the hosts of *Ur. unguiculata* from the guts of blood-engorged flies. The hosts were identified as anurans, and a similar host preference is known for other members of the genus *Uranotaenia* [7, 8]. Commonly encountered species of anurans at BSI during the study period included *Pelophylax* sp., as well as *Hyla arborea* (Linnaeus, 1758) (Anura: Hylidae), *Bufo bufo* (Linnaeus, 1758) (Anura: Bufonidae) and *Bombina bombina* (Linnaeus, 1761) (Anura: Discoglossidae) (*via* visual encounter during trap setup). It is unknown if *Ur. unguiculata* takes blood from animals other than frogs. Previous attempts to identify the hosts of *Ur. unguiculata* have relied on serological testing, wherein amphibian antiserum was not used or was unavailable, and the host identities were determined to be from reptiles [10] or a horse [11]. It has been reported that *Ur. sapphirina* (Osten Sacken, 1868) (Diptera: Culicidae), a Nearctic species, may also feed on mammals in addition to amphibians [7, 29]. Furthermore, landing captures and captures from host-baited traps suggest that *Ur. unguiculata* may be attracted to mammals including humans [9, 11, 30] but not birds [3]. The relatively low efficiency of common mosquito collection methods (e.g. CO_2_-baited light traps) for the collection of *Ur. unguiculata* has left many gaps in the knowledge of the behaviors of this species.

Efficient collection methods for a mosquito species reflect its host preference and common methods (e.g. CO_2_-baited light traps) are designed to collect mosquitoes based on foraging behavior during appetential flight [31]. For example, trap height is an important factor for collecting ornithophilic *versus* mammal-biting mosquitoes [32–34]. Sebesta et al. [3] collected *Ur. unguiculata* in 1 m high CO_2_-baited CDC light traps, and none in 5-m high traps nor in pigeon-baited traps. We reasoned that *Ur. unguiculata* foraging preference would reflect host behavior, and therefore placed our traps near the water surface (0.5 m height). Although we did not compare the importance of trap height to collection efficiency of *Ur. unguiculata* directly, this trap placement was an improvement compared to other published records of *Ur. unguiculata* in the same trapping locales [5, 26] as well as in the broader surrounding biogeographical regions [35] using common mosquito collection methods. Importantly, we collected both male and female *Ur. unguiculata*; the efficient collection of males to our knowledge has not yet been reported.

Although we provide evidence that *Ur. unguiculata* females are attracted to sound, it is unknown if sound is used exclusively to locate hosts. The host species identified here, *Pelophylax* sp. and *B. bombina* were the only two species of anurans heard calling during the collection period. Sound attraction is known from *Ur. lowii* Theobald, 1901 (Diptera: Culicidae), a Neotropical species of mosquito [17], and several species of *Uranotaenia* from Japan [8]. Acoustic location of hosts by hematophagous dipterans is best known from the Corethrellidae (Wood & Borkent 1989), a family closely related to mosquitoes which feed on frogs [36–38]. The sound of the calling male *D. gratiosus*, a Nearctic species of frog, is attractive to *Ur. lowii* as well as to Neotropical and Australian corethrellids. Therefore it has been hypothesized that acoustic location in corethrellids is performed by a sensory organ that detects frequencies that are similar to wing beat frequency (approximately 420–450 Hz), which match the dominant frequency of the call of *D. gratiosus* (Additional file 1: Figure S1) [37, 39, 40]. The Johnston’s organ is known to be the auditory organ of mosquitoes, and is used to identify the wing beat frequency of conspecifics during mating, though may be sensitive up to 2 kHz in some species [41, 42]. Further studies on acoustic location and sound preference of *Ur. unguiculata* are underway, and the attractiveness of native anuran calls will be evaluated.

Knowledge of mosquito host feeding behavior is very important for understanding the epizootic potential of arboviruses. The presence of WNV-lin. 4c nucleic acid was recently reported in *Ur. unguiculata* from the study location here, as well as other sites in central Europe [4–6]. Here we provide evidence that *Ur. unguiculata* are feeding on *Pelophylax* sp. at a site where WNV-lin. 4c was detected in pools of conspecifics, including evidence that suggests the mosquitoes are both infected with the virus and are capable of transmitting the virus. Taken together with the detection of virus nucleic acid present in both *Ur. unguiculata* and also frogs (*P. ridibundus*) collected from a site in southern Russia [14], it is likely that this virus is maintained in a frog-mosquito transmission cycle. The first identification of WNV-lin. 4 was from a *Dermacentor marginatus* (Sulzer, 1776) (Acari: Ixodidae) tick in the Volgograd region, southern Russia, in 2003 (WNV-lin. 4a; GenBank: AY277251) [12], and a similar virus has also been isolated from *Cx. pipiens* in Spain 2011 (WNV-lin. 4b; GenBank: GU047875) [13]. In sum, there is further support for WNV-lin. 4c being ecologically and genetically distinct from other WNV lineages [43]. Since WNV-lin. 1 and WNV-lin. 2 are known pathogens to humans and animals, it is important to understand the epizootic potential of the WNV-lin. 4c. Initial efforts have been successful in isolating this virus on C6/36 cells, and future efforts should characterize the pathogenicity of the virus. Additionally, the vector status of *Ur. unguiculata* for WNV-lin. 4c must be determined in controlled experiments.

The genus *Uranotaenia* has few reports of infection with arboviruses. Eastern equine encephalitis virus was discovered in *Ur. sapphirina* in the southern USA [44], and a cyprovirus (*Reoviridae*) has also been isolated from this species [45]. A novel flavivirus, Nounane virus, was reported from *Ur. mashonaensis* Theobald, 1901 (Diptera: Culicidae) in Côte d’Ivoire [46]. Nounane virus bears closest similarity to Barkedji virus, a virus found in *Cx. perexiguus* Theobald, 1903 (Diptera: Culicidae) mosquitoes in Israel [47], and Nhumirim virus, a virus found in *Cx. chidesteri* Dyar, 1921 mosquitoes from Brazil [48]; the vertebrate host is unknown for these viruses, although all are genetically similar to mosquito-borne zoonotic flaviviruses and not to insect-specific flaviviruses. Herein we report a potentially endogenous flavivirus-derived genomic sequence, identical to the sequence reported by others in *Ur. unguiculata* [15], possibly reflected an ancient association between *Uranotaenia* and flaviviruses [49, 50]. In addition, we isolated and sequenced the genome of an alphamesonivirus which is widespread in the population at BSI. Another member of the family *Mesoniviridae*, Meno virus, was isolated from Côte d'Ivoire, 2004, where it was found in *Ur. chorleyi* Edwards, 1936 (Diptera: Culicidae) along with Cavally virus, the first insect-associated nidovirus [27, 51]. Much remains unknown about this newly discovered family of insect viruses, which are within the *Nidovirales* [27, 28]. Future efforts will further characterize the virus isolate reported herein, and the presence of this or another alphamesonvirus within other mosquito species in the region will be tested.

In general, the comparatively low efficiency of collection and lack of human-biting behavior have likely contributed to the gaps in knowledge about this mosquito species in the Western Palaearctic. The optimized sampling conditions reported here increased trapping success compared to previous methods. This will aid in future studies of the habits of this elusive mosquito, and the transmission of arboviruses.

## Conclusions

Improved collection techniques have yielded this first definitive report of the hosts of *Ur. unguiculata* using molecular methods, and have revealed the presence of a novel insect-specific flavivirus. The mosquitoes take blood from frogs, *Pelophylax* spp*.*, and may transmit WNV-lin. 4c to frogs, although vector competence has not yet been established. It was discovered that *Ur. unguiculata* are attracted to sound, potentially as a method of acoustic location of hosts. Further research will focus on the ecological association between the mosquitoes and their hosts, as well as the transmission ecology of WNV-lin. 4 and the newly-described *Alphamesonivirus 1* isolate.

## Additional file

Additional file 1: **Figure S1.** Frequency spectrogram of a single vocalization of *Dryophytes gratiosus* (Anura: Hylidae) used as an attractant in mosquito sound traps. The grayscale spectrogram was generated in Raven Lite version 1.0 [52]. The spectrogram displays sound frequencies over time where darker pixels indicate relatively louder tones. The dominant frequencies of the call are approximately 450 and 2000 Hz, with harmonics above and below the 2 kHz tone. Charif, RA, DW Ponirakis, and TP Krein. 2006. Raven Lite 1.0 User’s Guide. Cornell Laboratory of Ornithology, Ithaca, NY. (TIF 66 kb)

Additional file 2: **Table S1.** Primers used to generate a nearly full-length sequence of a newly-described *Alphamesonivirus 1* (*Mesoniviridae*) in *Uranotaenia unguiculata* mosquitoes found in Austria. (DOCX 20 kb)
