# Supplementary figures and images for: Uranotaenia unguiculata Edwards, 1913 are attracted to sound, feed on amphibians, and are infected with multiple viruses
Source: Parasit Vectors. 2018 Aug 6;11:456. doi: 10.1186/s13071-018-3030-2 (PMC6090806; doi:10.1186/s13071-018-3030-2)

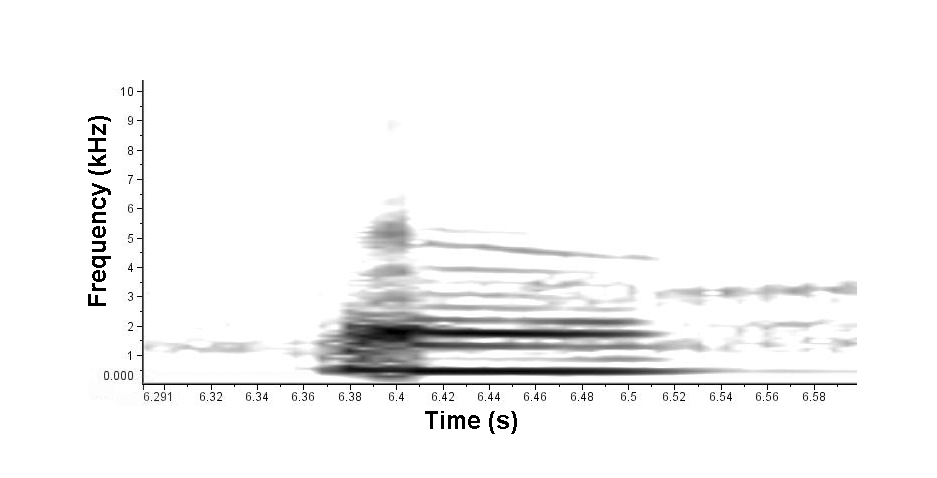

Supplement: Supplementary file 1 — Figure S1. Frequency spectrogram of a single vocalization of Dryophytes gratiosus (Anura: Hylidae) used as an attractant in mosquito sound traps. The grayscale spectrogram was generated in Raven Lite version 1.0 [52]. The spectrogram displays sound frequencies over time where darker pixels indicate relatively louder tones. The dominant frequencies of the call are approximately 450 and 2000 Hz, with harmonics above and below the 2 kHz tone. Charif, RA, DW Ponirakis, and TP Krein. 2006. Raven Lite 1.0 User’s Guide. Cornell Laboratory of Ornithology, Ithaca, NY. (TIF 66 kb) [file 13071_2018_3030_MOESM1_ESM.tif]
