# Supplementary material for: Uranotaenia unguiculata Edwards, 1913 are attracted to sound, feed on amphibians, and are infected with multiple viruses
Source: Parasit Vectors. 2018 Aug 6;11:456. doi: 10.1186/s13071-018-3030-2 (PMC6090806; doi:10.1186/s13071-018-3030-2)
Supplement: Supplementary file 2 — Table S1. Primers used to generate a nearly full-length sequence of a newly-described Alphamesonivirus 1 (Mesoniviridae) in Uranotaenia unguiculata mosquitoes found in Austria. (DOCX 20 kb) [file 13071_2018_3030_MOESM2_ESM.docx]

**Additional file 1: Table S1.** Primers used to generate a nearly full-length sequence of a newly-described *Alphamesonivirus 1* (*Mesoniviridae*) in *Uranotaenia unguiculata* mosquitoes found in Austria.

Camp, et al. “*Uranotaenia unguiculata* Edwards, 1913 are attracted to sound, feed on amphibians, and are infected with multiple viruses.”

| **Primer Pair** | **Name** | **Sequence (5'->3')** |
| --- | --- | --- |
| 1 | Meso273F | ACAGAGGCTATATTAGTGCTCA |
| 1 | Meso1127R | GTGGCGTCATCTAGCTCGTA |
| 2 | Meso1004F | GCTTACCCACATCTCGGCTT |
| 2 | Meso1933R | CTGTAGCAGTGGTGGCAAGA |
| 3 | Meso1913F | GTCTTGCCACCACTGCTACA |
| 3 | Meso2846R | TGGTTAAGCGTGCCCAAAACA |
| 4 | Meso2636F | AGCTCACACATCACGACTTCA |
| 4 | Meso3570R | GAAACCAATGGCATCGCTCA |
| 5 | Meso3546F | TGAATTGAGCGATGCCATTGG |
| 5 | Meso4559R | ATTGAGGGGTTTGATGCTGC |
| 6 | Meso4427F | ACTGTCGCCGCTTCAACTTA |
| 6 | Meso5588R | TAGAAGGCTGGGTGTGTGTG |
| 7 | Meso5407F | GGGACCAAAAGCTTCGAAACC |
| 7 | Meso6523R | TCACGCGTACCAGATTGTTCA |
| 8 | Meso5884F | ATTAAACCCTACGCCGCCTT |
| 8 | Meso6955R | GCGTGGCAGTGCAAGTAAAT |
| 9 | Meso6765F | CTGACCTGGAATTGGACCCC |
| 9 | Meso7874R | AGAGTACGAGCCCACCTTGA |
| 10 | Meso7734F | ACACCGTTAATCATGCCACCA |
| 10 | Meso8678R | GTGTCATCAAACTTGGCGTCC |
| 11 | Meso8637F | AGTTTGAATTGGCAGTTGAGGC |
| 11 | Meso9667R | AGGACAGTTTGGCTTGCCAT |
| 12 | Meso9570F | CCGGCTCAGTTTCACCTACA |
| 12 | Meso10772R | GCATTGCGGTATACATGCGT |
| 13 | Meso10662F | CACATCGACGCTATCCAACG |
| 13 | Meso11581R | TATTGCATAGGTCGGGTGTG |
| 14 | Meso11409F | TTACTGTACACCACTGGCCG |
| 14 | Meso12349R | CCGGGGTTTTGTATAGTGCG |
| 15 | Meso12309F | CACACCATCACACCAATCTGC |
| 15 | Meso13557R | GCCTGCTATACGTCGTTTGC |
| 16 | Meso13334F | CACCAACAACGCTAAAGACCA |
| 16 | Meso14189R | TCATCAAGTGGGTCGTGTGG |
| 17 | Meso13915F | CCACAAGGCCGACACACTTA |
| 17 | Meso14621R | AGACATAAGCCAGTGGTCCC |
| 18 | Meso14488F | ACCTGTGATTCCGAAGAGCA |
| 18 | Meso15556R | ACTCAGCCCTCAGTTCATGTG |
| 19 | Meso15403F | CGGAGCGACTGAAGACGTAA |
| 19 | Meso16256R | GAGGATTGCATTGAAGGCGT |
| 20 | Meso16349F | GCACGCACCACATAGTTTCA |
| 20 | Meso17184R | AGCCGTGACGAGTTGTTGAA |
| 21 | Meso16999F | GCTAAAGTGCTGGGAGGTGA |
| 21 | Meso18178R | AGTCTTCGTCAGCTGTGGTG |
| 22 | Meso18024F | ACATCCCTCCCTACGTGTCA |
| 22 | Meso18725R | ACCAAAAGTGTGACGCTAGC |
| 23 | Meso18851F | AAAACCGCCTGAGTTTAGTT |
| 23 | Meso19692R | CGGGGTCCTAATGCTTCACTA |
